# Empirical and Comparative Validation for a Building Energy Model Calibration Methodology

**DOI:** 10.3390/s20175003

**Published:** 2020-09-03

**Authors:** Vicente Gutiérrez González, Germán Ramos Ruiz, Carlos Fernández Bandera

**Affiliations:** School of Architecture, University of Navarra, Pamplona 31009, Spain; gramrui@unav.es (G.R.R.); cfbandera@unav.es (C.F.B.)

**Keywords:** building energy models (BEMs), calibration, sensors, energy simulation, sensors saving, calibrated model validation, methodology

## Abstract

The digital world is spreading to all sectors of the economy, and Industry 4.0, with the digital twin, is a reality in the building sector. Energy reduction and decarbonization in buildings are urgently required. Models are the base for prediction and preparedness for uncertainty. Building energy models have been a growing field for a long time. This paper proposes a novel calibration methodology for a building energy model based on two pillars: simplicity, because there is an important reduction in the number of parameters (four) to be adjusted, and cost-effectiveness, because the methodology minimizes the number of sensors provided to perform the process by 47.5%. The new methodology was validated empirically and comparatively based on a previous work carried out in Annex 58 of the International Energy Agency (IEA). The use of a tested and structured experiment adds value to the results obtained.

## 1. Introduction

With the continuing challenges posed by climate change, a growing number of countries around the world are implementing measures to reduce energy consumption and greenhouse gas emissions. The increasing deployment of energy efficiency measures in the building sector provides an important avenue for reducing energy demand and carbon dioxide emissions, even generating new energy production and distribution facilities. The global market for energy efficiency in the construction sector will grow from USD 68.2 billion in 2014 to USD 127.5 billion in 2023 according to Navigant Research [1]. The high energy consumption of both commercial and residential buildings in developed countries, around 40% [2], necessitates a reduction in energy consumption. For this reason, energy-efficient construction is now a key factor in energy policies at all levels [3].

Energy simulation has become a powerful tool supporting the design of new buildings and in proposing Energy Conservation Measures (ECM) in existing buildings. It is used in the application of the predictive model control (MPC) techniques [4,5,6,7,8,9], in actions for the operational optimization of Heating Ventilation Air Conditioning (HVAC) [10,11], in economic strategies [12,13,14,15], and in the optimization of cost-effective building refurbishment [16,17]. In terms of building energy models (BEMs), Hence and Lamberts [18] differentiated between the following types depending on the physical relevance of the parameters:

- White box models are based on a physical models with exclusively physically meaningful parameters. This can provide the most detailed building performance characteristics that can be applied in energy prediction for demand response applications or establishing baseline models for ECM performance, among others.
- Black-box models are mathematical models constructed from training data. They typically lack physical meaning in their mapping of input parameters. This models can be developed in short time although frequent re-training can be required to adjust small changes in a building.
- Grey box models is a hybrid of data driven and physics based models where, coefficients of the equations from physics based models are learned using data. This approach allows to capture the dynamics of the buildings more effectively as compared to pure data driven approach [19,20].

On the basis of this classification, it is necessary to specify the number of parameters involved in the model that are to be adjusted to those of the real building. This process is called calibration. According to the American Society of Heating, Refrigerating and Air-Conditioning Engineers (ASHRAE) [21], the parameters can be adjusted based on two main concepts: (1) forward modeling, where all the necessary model parameters are known, but this is limited in use because the assumption of parameters knowledge can be only valid for some.

The term forward implies that there is no coupling between simulation and measured data. The models have internal engines for calculus that represent the thermal principles, and the parameters are represented by material characteristics. (2) Inverse modeling, where some or all the parameters are estimated based on in situ measurements.

The calibration process of a building basically consists of bringing the energy model closer to reality [22,23]. The results are evaluated by comparing the measured data curves with simulated data, in which is called uncertainty analysis, where a statistical index measured how well the agreement is represented. The simulation can have errors, even when the real and measured data coincide [24]. There are many sources of uncertainty, e.g., the weather [25,26,27] or the occupation [28], in this paper the center is the envelope parameters. Many degrees of liberty in the calibration process can produce good agreement with the measured data [29], but have an error in the individual datum [30,31]. Standard methodologies are lacking to produce calibrated BEMs. ASHRAE Guidelines 14 and International Performance Maintenance and Verification Protocol (IPMVP) [24,32] have established uncertainty analysis (Normalized Mean Bias Error: NMBE, Coefficient of Variation of Mean Square Error: CV(RMSE), and Square Pearson Correlation Coefficient: R${}^{2}$) to quantify when a model is calibrated, but no standard pattern exists of how these models can be produced or how they can be empirically validated. Therefore, a validation process of the calibration methodology is essential; otherwise, energy savings [33], diagnostic control of the installations, or MPC, among others, will be uncertain.

In relation to calibration techniques they are manual and automated. Regarding manual processes, Soebarto [34] proposes calibration with graphical and statistical analyses making changes in the input parameters, whereas Weil et al. [35] performed an analysis to investigate the signatures of different parameters on the heating and cooling energy consumption of typical air handling units(AHUs) in graphic format. With reference to automated processes based on black box models, Heo et al. [36] perform a calibration using Bayesian approach, Manfren et al. [37] calibrate through meta-models, O’Neill and Eisenhower [38] use sensitivity analysis with meta-models, and, finally, Sadeghian Broujeny et al. implementated a data-driven machine learning-based identification of the building’s heating living space. Black Box models are fast in execution; but, among the limitations, they are the complexity to establish models with multiple thermal zones.

The white box models, in turn, can be divided into: reduced order models [39,40] (RC models) and dynamic models based on complexity [41,42]. RC models are often used when MPC solutions are to be proposed due to their high calculation speed, whereas dynamic models, based on high-fidelity physics, can provide a more accurate analysis of energy performance [23]. However, they require a large amount of input data, some of which are difficult to obtain, resulting in large uncertainty [36,43,44,45].

High quality BEM will be an important element in the smart grids in the future. SmArt BI-directional multi eNergy gAteway (SABINA) is a European Union (EU)-funded H2020 research and innovation project [46] that aims to develop new technology and financial models to connect, control, and actively manage generation and storage assets to exploit synergies between electrical flexibility and the thermal inertia of buildings. Flexibility needs to be added to Europe’s power system to accommodate an increasing share of variable power generation from renewable sources. The SABINA project responds to this need by targeting the cheapest possible source of flexibility: the existing thermal inertia in buildings and the resulting coupling between heat and electricity networks. The use of thermal inertia as storage capacity is known as a power to heat (P2H) solution [47,48]. High quality models (calibrated) are constructed on the basis of the success of P2H implementation to offer services to the grid [6,8].

Neymark et al. indicated that there are three ways of evaluating a whole-building energy simulation program: (1) empirical validation, which compares simulated data with monitored data from a real building or experiment; (2) analytical verification, which compares simulated data from verified numerical models; and (3) comparative testing, which compares a program with itself or to other programs or techniques within the same program [49]. In this paper, an empirical and comparative test have been followed out.

Empirical validation must be used if an absolute standard of truth is to be established (in comparison with simulation results with a perfectly performed empirical experiment) [50]. Measurement error and uncertainty must be considered when constructing the model. Careful work is required to reduce these unknowns (geometry, materials, infiltrations, ground, etc.). The National Renewable Energy Laboratory (NREL) divides empirical validation into levels depending on the degree of control over possible error sources [51].

The comparative test examines the results obtained in the model with itself or with other energy models of the same building. This last test does not generate input uncertainties and can be applied regardless of the complexity of the model, as many comparisons can be performed as possible and it is cheap and fast. However, there are no absolute truth inputs, and only statistically-based acceptance ranges are possible. Among the techniques for testing a calibration methodology applying empirical, analytical, and comparative validation, there is the Building Energy Simulation Test for Existing Homes (BESTEST-EX) [52]. Additionally, the ASHARE 140 standard covers the analytical and comparative validation test [53]. The combination of analytical and comparative validation techniques reduces uncertainty in simulation processes where adjustment periods are short within the space in which they can be compared. By combining these options, model errors can be fixed and a more suitable solution can be reached [54,55,56].

In this study, based on previous calibration knowledge [57,58], a novelty in the methodology has been implemented with a reduced number of parameters (thermal inertia, infiltration, and thermal bridges), which simplified dramatically the technical complexity of the process. The calibrated BEM produced complies with standards criteria of ASHRAE and International Performance Measurement and Verification Protocol (IPMVP), in temperature with hourly CV(RMSE) below 2%, in energy with hourly CV(RMSE) between 9 to 30% and in both cases with R2 above 90%. The new methodology was validated empirically and comparatively based on a previous work carried out in Annex 58 of the International Energy Agency (IEA). Ramos et al. [57] and Bandera et al. [58] published a BEM calibrated with genetic algorithm producing hourly CV(RMSE) below 4% and R2 above 90% in temperature, but no information about energy was given; in both papers the number of parameters used for calibration were fifteen, neither of them were empirically validated. Tahmasebi et al. [23] produced a calibrated energy model of an office building with an hourly CV(RMSD) of 2.35% and a R2 of 88% in temperature without information on energy with six parameters. Raftery et al. [59] presented an hourly CV(RMSE) of 8.72% in energy consumption with more than 20 parameters involved and without information about thermal zone temperature. Chaudhary et al. [60] in the Autotune project reduced the hourly CV(RMSE) in energy to less than 5% by using 60 parameters. Chong et al. proposed a bayesian calibration framework in an EnergyPlus model of the cooling system of a ten story office building located in Pennsylvania with hourly energy CV(RMSE) of 6% by using ten parameters and without information about temperature in building thermal zones. Cacabelos et al. performed a similar work with low quantity of envelope parameters involved, with an hourly CV(RMSE) of 5% in temperature but with a monthly CV(RMSE) of 4%.

The remainder of this paper is structured as follows: Section 2 explains the methodology used for the validation of the calibration process and provides an analysis of the sensors provided and the sensors used to perform the analysis. Section 3 details the analysis of the results. Using uncertainty indices, the results were evaluated in both energy and temperature, and the final values of the objects used in the process are reported. The paper finishes with the conclusions, which are discussed in Section 4.

## 2. Empirical Validation and Comparative Test Methodology

In this paper, a envelope calibration technique was validated using empirical and comparative tests with a case study provided by Annex 58 [61]. High quality calibration was achieved by considerably reducing the number of parameters and sensors used in the original experiment (by 47.5%), using 42 out of the 80 sensors. This simplification does not reduce the quality of the calibration process because the as-build data are taken as a base model. The method of measuring the calibration quality of the models was widely studied in our previous scientific articles [62,63,64] and in other studies [65]. This novel methodology reduces the uncertainty of the error since it is able to find the optimal adjustment with a minimum number of parameters that fit the curve of the simulated data with respect to measured data. The methodology captures the building thermal dynamics and therefore quantifies the available thermal mass. Thermal mass plays an important role in the heat transfer within a building [66], but many simulation programs only consider the thermal mass of the envelope, neglecting what may exist inside the building (furniture, partitions, books, etc.) [67]. This means that thermal zones are treated as empty air-filled spaces [68,69,70]. Similarly, dynamic infiltration is another parameter that is difficult to obtain due to the size of the leaks in the building, the climatic conditions, the permeability of the facade, air flows, etc. They are complex to measure [71].

This methodology tries to adjust these values using real data based on the high quality models obtained. None of the seven tests and trials in the buildings conducted with the aim of providing useful information for the process were used. As such, the uncertainty caused by the input elements was decreased and cost-effectiveness was increased. We focused on twin houses in the German town of Horzkitchen where all the as-built information was available, so that the energy model was constructed with less uncertainty. The original experiment was performed by another 21 research groups with different techniques and simulation engines. To avoid commercial competence, there was no information on how to connect the model characteristics and results with specific simulation software. In this paper, all the information about the parameters estimation of the model is provided so the experiment can be reproduced and validated. As far as the authors are aware, this is the first time this experiment has been used for empirical validation out of the Annex contest.

The simulation engine was EnergyPlus 9.2 [41], and the optimization-based calibration tool was JePlus + EA [72].

### 2.1. Selection of Data Provided by Annex 58 for Empirical Validation

The data used in this project were part of work completed by Annex 58, and all the information is available on the Internet [73]. The process of calibration started by feeding the base model the data collected from the sensors. Table 1 shows the sensors that were placed in the buildings. Two types of sensors can be distinguished: those that refer to the building (indoor temperature, energy consumption, etc.) and those that refer to weather conditions (exterior temperature, solar radiation, wind speed, etc.). In addition to the sensors, several analyses and tests were conducted in the houses to obtain additional information that may be useful for the empirical test:

- In the area of windows, Window 6.3 software was used. We used publicly available computer software that provides a versatile heat transfer analysis method consistent with the updated rating procedure developed by the National Fenestration Rating Council (NFRC), which is consistent with the ISO 15099 standard. With Window 6.3, the optical properties of house glass were calculated. The Fraunhofer IBP Institute (Institute for Construction Physics) calculated the absorption capacity of the blinds.
- The transmittance values of the thermal bridges were calculated using TRISCO and THERM software. The latter is state-of-the-art computer software that performs a two-dimensional conduction heat transfer analysis based on the finite element methodology. The Fraunhofer IBP Institute provided the U values of the thermal bridge for windows similar to those built in the houses.
- For ventilation, apart from the sensors used, the PHluft program of the Passive House Institute was used. This is a free program that calculates the heat transfer between the ventilation ducts and the indoor environment.
- The infiltration of the houses was obtained by the use of blower doors. A blower door is a machine used to measure the hermeticism (air tightness) of buildings. It can also be used to measure the flow between built areas, to test the tightness of air conductors, or to help physically locate air escape sites in the building envelope. A total of five blower doors were used between the two houses. They were applied throughout the house and in the rooms that were part of the experiment.
- For the ground, the reflection of the short wave ground was measured. Reflectivity measurements were recorded on both asphalt and gravel and the ground temperature was recorded at various depths: 0, 0.05, 0.1, and 0.2 m.

To perform the novel calibration procedure, all the information was deeply analyzed. The sensors required for the process were listed and are highlighted in grey in Table 1. This selection shows that the number of sensors was significantly reduced (42 out of 80, a 47.5% reduction) and that the sensors used were unobtrusive. Regarding analysis and testing, none of the data provided were used to conduct the experiment. These findings show that the proposed methodology is cost effective.

### 2.2. The Buildings

Once the data used to obtain the energy model were analyzed and validated, the geometry of the buildings were drawn. To do this, OpenStudio [74] was chosen and the generated volume was exported to EnergyPlus and JePLUS+EA to perform the optimization-based calibration process. EnergyPlus is an open source building energy simulation engine developed by the DOE (U.S. Department of Energy), tested and validated through analysis and benchmarking.

**Figure 1:**
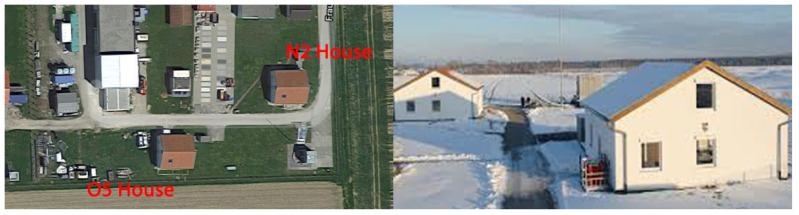
External views of twin Houses (N2 and O5). Holzkirchen, Germany.

**Figure 2:**
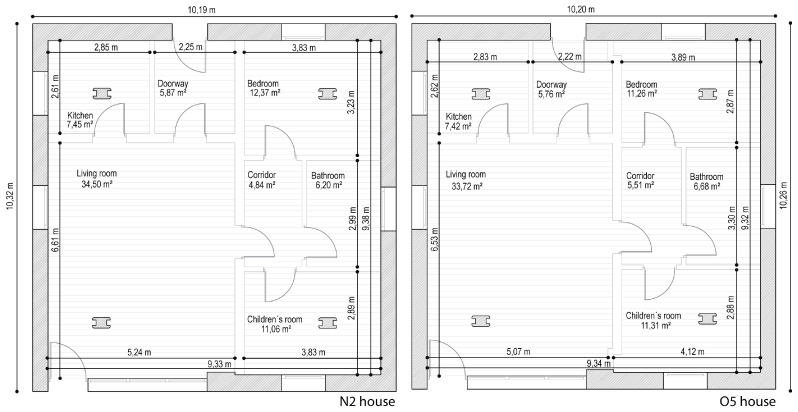
Plans of the houses.

This study was conducted on two prototype houses (N2 and O5 houses) in the German town of Holzkirchen, south of Munich (Figure 1). The houses are twins and are located in a flat area without any buildings that could cast shadows on them in the summer period. They have three floors: basement, ground floor, and attic. The experiment focused on the ground floor, which included a living room, kitchen, entrance, bathroom, corridor, and two bedrooms (Figure 2). It has a free height of 2.495 m.

Once the geometry of the buildings was drawn, the physical properties and the thermal load of the buildings were introduced.

The documentation provided by the Annex 58 test includes all the materials and construction elements of the houses, detailed information about the windows (windows glass, frames, and dividers), and the thermal bridges.

For heating, the houses are equipped with Dimplex AKO K 810/K 811 electric radiators with an estimated rapid response time of 1 to 2 min with a radiative-convective effect of 30%/70%.

It was reported that the ventilation of the houses is mechanical. The supply and extraction points are located on the ceiling. Constant ventilation is provided during the whole test period, which introduces 120 m^3^/h of air through the living room ceiling and extracts 60 m^3^/h through the bathroom and 60 m^3^/h through the child’s room.

### 2.3. Experimental Design and Calibration Process

In order to carry out the study, we ensured that the heat flow in the houses guaranteed the possibility of constructing satisfactory energy models with reasonable quality. To achieve this energy activation, the designers of the original experiment subjected the houses to three types of sequences: internal steady-state temperatures, a sequence of pseudo-random heat injections, and a period of free oscillation.

The experiment was conducted in the months of August and September 2013 (from 1 August to 26 September) because the houses were only available on those dates. Although these are summer months, heating was used in the exercise. During this time, the houses underwent five periods of energization to reflect, among other things, the common conditions of the buildings and to ensure that the dynamic response was tested (Table 2).

- Period 1: In this first period, the aim was to achieve identical and well-defined starting conditions for both houses. To do this, they were heated to 30 °C for three days.
- Period 2: During the following seven days, the interior temperatures were kept constant at 30 °C using the building’s control system. For the experiment, indoor temperatures were provided as inputs to the mode, and the energy needed by the HVAC system to achieve those temperatures was requested.
- Period 3: In this period, a Randomly-Ordered Logarithmic Binary Sequence (ROLBS) was implemented for the activation of the living room radiator (the rest of the radiators in the rooms were turned off, thus increasing the interaction between the units). This sequence was developed in the EC COMPASS project. The ROLBS, which aims to cover all relevant frequencies with the same weight, is a signal in which the on and off periods are chosen at logarithmically equal intervals and shuffled in a quasi-random order. This random sequence ensures that there is no relationship between the heat input by the HVAC system and the solar gains. This phase lasted two weeks with heat inputs ranging from 1 to 90 h. The power of the radiator was limited to 500 W. During this stage, the energy consumed by the radiator was offered and the energy model was asked to predict the interior temperatures of the rooms.
- Period 4: After Period 3, the thermal load of the houses was reset so that in the following period, both houses started with the same temperature and internal energy conditions. To achieve this, over 7 days, a constant temperature of 25 °C was introduced. As in Period 2, the indoor temperatures were provided so that they could be entered into the energy model and were asked to predict the energy involved in raising the indoor temperature to 25 °C.
- Period 5: This was the last stage of the experiment. During this time, no energy was introduced into the buildings; they were left in free oscillation. The energy model was asked to reproduce the indoor temperatures using only input of energy provided by the external weather.

After developing the base BEM, the new calibration process began to find the best adjustment of energy and indoor temperatures for each of the design periods. A script programmed in EnergyPlus run-time language was developed. This script transferred the measured temperature to the model as a set-point in Period 2 and 4. Similarly, real energy consumption was input to the model as an internal load in Period 3 and 5. Under normal circumstances a temperature sensor per thermal zone is enough but if available more information can be added. Energy consumption is not essential in this process because the calibration can be performed in a free float period.

**Figure 3:**
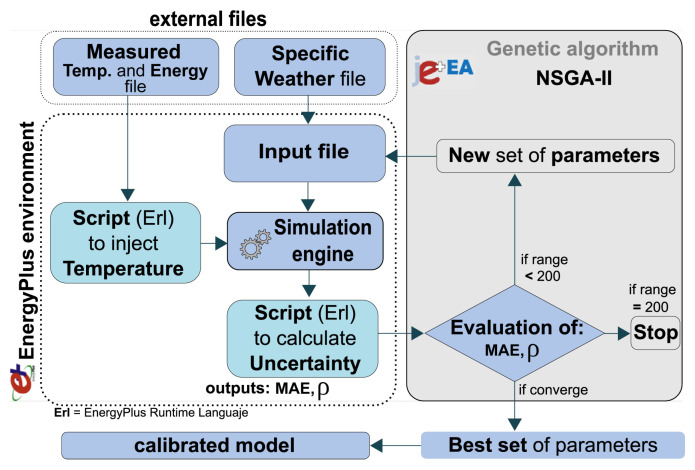
Calibration environment with genetic algorithm.

The calibration procedure represented in Figure 3 was repeated for the different periods described above. The methodology is similar to an optimization process but the objective function is related to the adjustment between real and simulated curves. The objective function of the calibration process, which will show the way to finding the model with the highest adjustment range, is composed of the uncertainty indices proposed by the contest. In this case, the Mean Absolute Error(MAE) and the Spearman rank correlation coefficient (ρ) were used, since they are the indices chosen in Annex 58, but other statistical indexes like CV(RMSE) are also possible. The genetic algorithm Non-Dominated Sorting Genetic Algorithm (NSGA-II) [75] was selected as the engine for tracking the best solution. The search space is based on the combinational probabilities of the parameters: capacitance, thermal mass, infiltrations, and thermal bridges, it is a 75% reduction compare with previous studies [57,58]. Another improvement implemented has been the introduction of the statistical index, in this case the MAE for each thermal zone, this makes the process faster to converge while in the previous studies a maximum of two objectives functions where in place for total energy and CV(RMSE) of the average temperature of the thermal zones.

In the proposed exercise, 21 research centers and universities from around the world participated. All members created their own energy models and reported their results for the different periods analyzed. The whole experiment was reported by Strachan et al. [61].

When considering the execution of the experiment, we decided to perform two exercises: the adjustment of the model for each period of analysis, which produced four models: one each for Periods 2 to 5 (Period 1 is the initialization of thermal conditions), and obtaining an energy model adjusted to all the proposed periods (called a unique model).

To perform an equitable evaluation of the data obtained by the different energy models and to qualitatively show the degree of agreement between the different periods and between the temperature and energy predictions, for the organization of Annex 58 test, we decided to use two evaluation metrics:

- To measure the magnitude adjustment, we used mean absolute error (MAE) in Equation (1), which is the measurement of the difference between two continuous variables, considering the two sets of data (some calculated and others measured) related to the same phenomenon:
  (1) $$MAE=\frac{1}{n}\sum_{i=1}^{n}\left|y_{i}-{\hat{y}}_{i}\right|,$$
  where $y_{i}$ and ${\hat{y}}_{i}$ are the real and simulated values, respectively, and n is the number of values in the test sample.
- To assess the level of correspondence of the form, we used Spearman’s rank correlation coefficient, ρ, using Equation (2). This coefficient is a measure of linear association that uses the ranges and the order number of each group of subjects and compares these ranges:
  (2) $$\rho =\frac{\sum_{i=1}^{n}\left(rg\left(y_{i}\right)-\bar{rg(y)}\right)\left(rg\left({\hat{y}}_{i}\right)-\bar{rg(y)}\right)}{\sqrt{\left(\sum_{i=1}^{n}{\left(rg\left(y_{i}\right)-\bar{rg(y)}\right)}^{2}\right)\left(\sum_{i=1}^{n}{\left(rg\left({\hat{y}}_{i}\right)-\bar{rg(y)}\right)}^{2}\right)}}.$$

**Figure 4:**
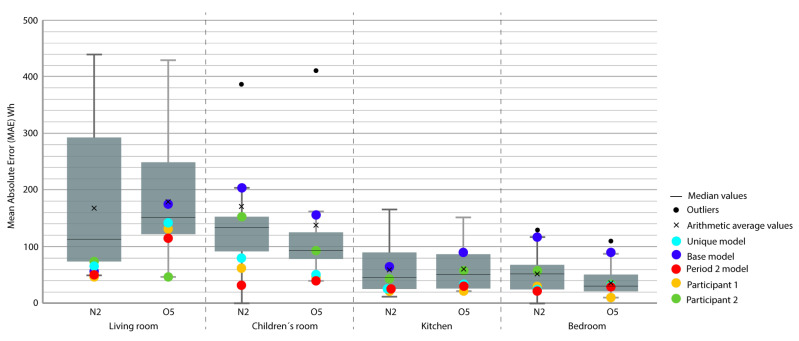
Energy mean absolute error (MAE) for Period 2 (fixed set point at 30 ${}^{{}^{\circ}}$C).

**Figure 5:**
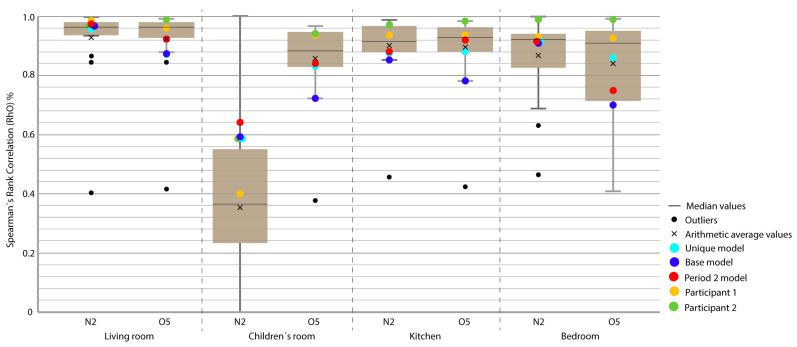
Energy Spearman’s rank correlation coefficient (ρ) for Period 2 (fixed set point at 30 ${}^{{}^{\circ}}$C).

**Figure 6:**
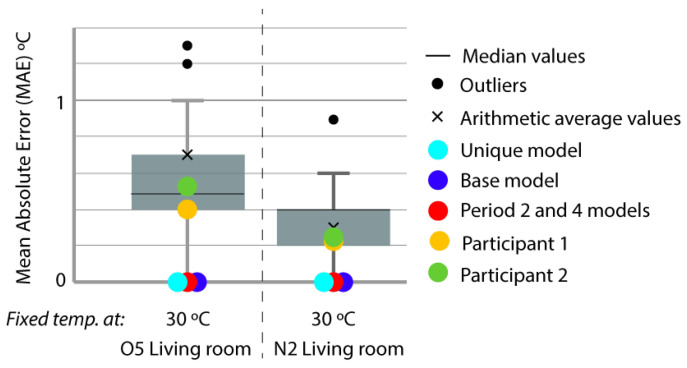
Temperature MAE for Period 2 (fixed set point at 30 ${}^{{}^{\circ}}$C).

**Figure 7:**
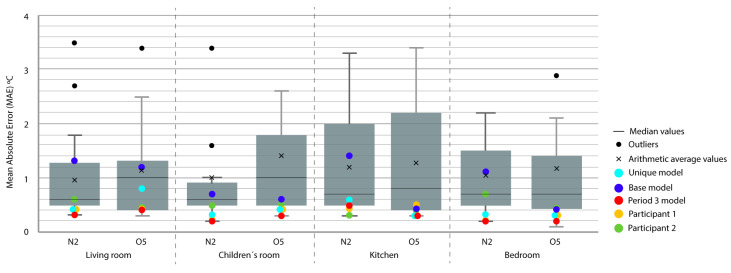
Temperature MAE for Period 3 (Randomly-Ordered Logarithmic Binary Sequence (ROLBS)).

**Figure 8:**
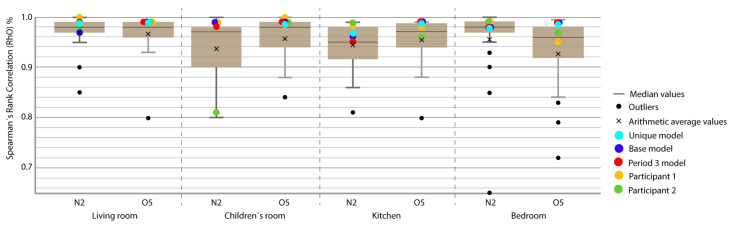
Temperature Spearman’s Rank Correlation Coefficient (ρ) for Period 3 (ROLBS).

**Figure 9:**
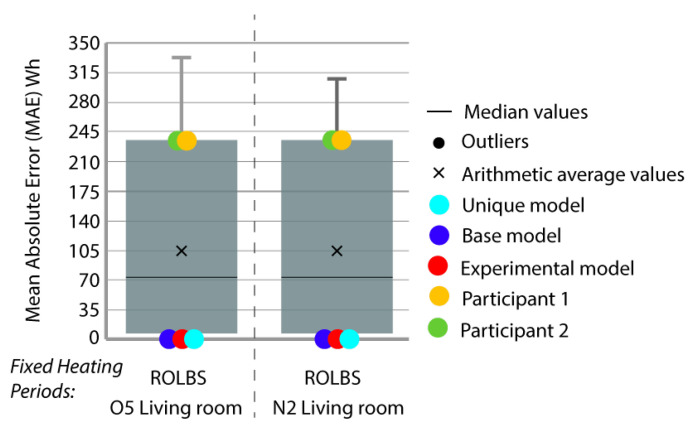
Energy MAE for Period 3 (ROLBS).

**Figure 10:**
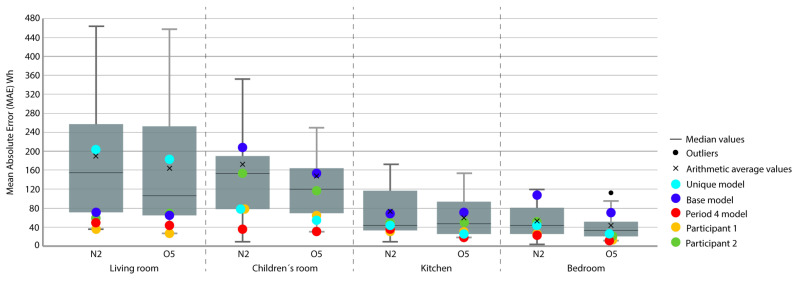
Energy MAE for Period 4 (fixed set point at 25 ${}^{{}^{\circ}}$C).

**Figure 11:**
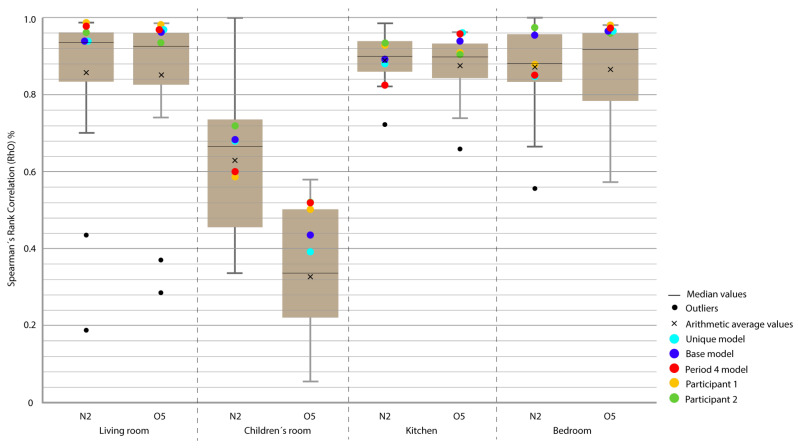
Energy Spearman’s Rank Correlation Coefficient (ρ) for Period 4 (fixed set point at 25 ${}^{{}^{\circ}}$C).

**Figure 12:**
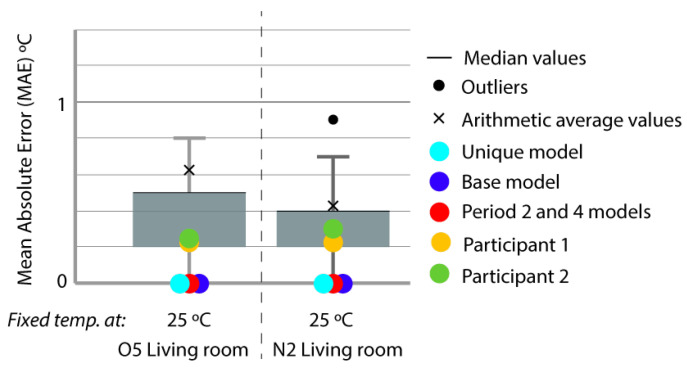
Temperature MAE for Period 4 (fixed set point at 25 ${}^{{}^{\circ}}$C).

**Figure 13:**
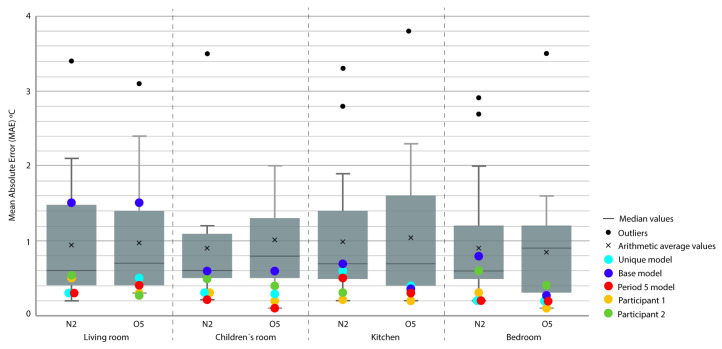
Temperature MAE for Period 5 (free oscillation).

**Figure 14:**
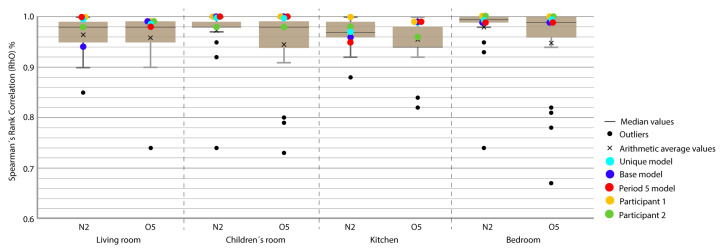
Temperature ρ for Period 5 (free oscillation).

For the representation of the obtained data, we chose the box plot because it is a standardized method that graphically represents a series of numerical data through its quartiles. The box plot shows the median and quartiles of the data at a glance and can also represent the outliers. The graphs represent the results of the 21 participants in the Annex 58 contest plus the experiment in this study. The graphs allow a clear and quick representation of the results obtained by all the participants, comparing them in an empirical and comparative way at the same time. (Figure 4, Figure 5, Figure 6, Figure 7, Figure 8, Figure 9, Figure 10, Figure 11, Figure 12, Figure 13 and Figure 14).

The models were evaluated using the energy and temperature data obtained from the living room, south bedroom (children’s room), kitchen, and north bedroom (Bedroom) of both houses, N2 and O5.

The standards for the evaluation of BEMs are provided by by ASHRAE, IPMVP, and the Federal Energy Management Program (FEMP). As such, the indexes proposed and recommended by those agencies for validation were included to this study: CV(RMSE), NMBE, and R^2^.

*CV*(*RMSE*) (Equation (3)) is the coefficient of variation of the mean square error, which is obtained by weighting the RMSE index by the average of the real values. This index considers the error variance as measured variability and is therefore recommended by ASHRAE guideline 14, by FEMP, and by IPMVP.

(3) $$CV\left(RMSE\right)=\frac{1}{\bar{y}}{\left[\frac{\sum_{i=1}^{n}{\left(y_{i}-{\hat{y}}_{i}\right)}^{2}}{n-p}\right]}^{\frac{1}{2}}.$$

NMBE (Equation (4)) is the normalized mean bias error. It is a modification of the MBE index that obtains more precise information about the adjustment between two values; in our case, this includes real values and those simulated by the energy models.

(4) $$NMBE=\frac{1}{\bar{y}\left(n-p\right)}\sum_{i=1}^{n}\left(y_{i}-{\hat{y}}_{i}\right).$$

The coefficient of determination R^2^ (Equation (5)) is the proportion of the variance in the dependent variable that is predictable from the independent variable. This coefficient is used to analyze how differences in one variable can be explained by a difference in a second variable. R^2^ is similar to the correlation coefficient, *r*. The correlation coefficient formula will tell you how strong a linear relationship exists between two variables.

(5) $$R^{2}={\left(\frac{\sum_{i=1}^{n}\left(y_{i}-\bar{y}\right)\left({\hat{y}}_{i}-\bar{\hat{y}}\right)}{\sqrt{\left(\sum_{i=1}^{n}{\left(y_{i}-\bar{y}\right)}^{2}\right)\left(\sum_{i=1}^{n}{\left({\hat{y}}_{i}-\bar{\hat{y}}\right)}^{2}\right)}}\right)}^{2}.$$

International Performance Measurement and Verification Protocol (IPMVP) states that a model can be considered calibrated if it achieves an NMBE of less than ±5% with a CV(RMSE) of less than ±20% on an hourly scale. ASHRAE and Federal Energy Management Program (FEMP) consider a model calibrated if its NMBE index does not exceed ±10% combined with a CV(RMSE) index of no more than ±30% (Table 3).

ASHRAE, in turn, recommends that models considered calibrated should not have an R^2^ index lower than 75%.

## 3. Analysis of Results and Discussion

To better understand the results, we introduced extra points into the box plot graphs (Figure 4, Figure 5, Figure 6, Figure 7, Figure 8, Figure 9, Figure 10, Figure 11, Figure 12, Figure 13 and Figure 14). The green and yellow points are the best results of the Annex 58 contest; they are labeled participant 1 and participant 2. Three extra points were shown: (1) in red, the best model obtained with the proposed methodology; (2) in dark blue, the base model created from the data provided by the developers of the experiment, without any previous calibration process; and (3) in sky-blue, the unique model that is better adjusted to all the proposed periods.

Through the box plot graphs, we attempted to answer the following research questions for each analyzed period: (1) By how much is the base model (dark blue) improved by the calibration process (red or sky-blue)? (2) How far is the distance between the unique models (sky-blue) and the best model for each period (red)? (3) What is the position of these models (red and sky-blue) in relation to the best models of the original experiment performed by Annex 58 (yellow and green)?

All the evaluation points of the models are represented with this type of graph, where the MAE is shown as the level of correspondence of the adjustment and the ρ of the shape, thus easily visualizing the results. The periods graphed are as follows:

Period 2 (fixed set point at 30 °C): The MAE (Figure 4) and ρ (Figure 5) of the comparison of the energy consumed by the energy model and the real energy consumed by the houses are shown for a set point of temperature fixed at 30 °C. Two and one outliers, respectively, are not shown in the figures because they were are not representative of the models.

In this case, the base model (dark blue) was improved by the calibrated models. In MAE and ρ, almost all the thermal zones are in Quartile (Q) 3 or Q4; after the calibration process, they are consistently in Q1 or Q2 (red and sky-blue). The distance between the red and sky-blue models is similar because they are systematically in the same quartile. The red model has a better position. In relation to the best models of the contest (green and yellow), in MAE, the red model is normally in the best position but in ρ, these model are better, especially the green model.

Figure 6 shows the MAE of the comparison of the indoor temperatures of the energy models with the interior temperatures of the houses in the period of constant set point (Period 2) in the living space.

In this period, the models created with the proposed methodology are: the Period 2 model, unique model, and base model, which do not generate any uncertainty when representing the real temperature of the building, since this temperature, provided by the authors of the exercise, is introduced in the model as input data. The models represent the real temperature and the energy it produces was analyzed in the previous figures. Our hypothesis was that the rest of the participants introduced error into the temperature to be able to better adjust the energy consumed by the model and thus be more similar to the real energy.

Period 3 (ROLBS): The MAE and ρ of the comparison of the indoor temperatures of the energy models and the real indoor temperatures of the houses, for a predefined energy, are shown in Figure 7 and Figure 8, respectively. Four outliers are not shown in Figure 8 because they were not representative of the models.

The base model performed slightly better than the previous case, MAE is Q3 or Q2 and near Q1 in some cases; ρ is Q2 or Q1. MAE was improved by the calibration process, in some cases from Q4 to Q1; the improvement in ρ was not so clear because most of the selected models were already Q1 or Q2. The distance between sky-blue and red was not significant, being never more than one quartile. In relation to the position of the models (red and sky-blue) in the contest, for MAE, red held consistently a leading position and for ρ, the amplitude of the boxes was smaller and all the models performed similarly. The kitchen was the place where red and sky-blue performed the worst.

Figure 9 shows the MAE when comparing the energy produced by the participants’ energy models with the real energy produced in the houses in this period (ROLBS) in the living room.

In Period 3, all the models that were calculated (red, sky-blue and dark blue) to validate the methodology consumed the energy that the real building demanded to reach the requested temperature. This means that they had no uncertainty in contrast to the rest of the participants who introduced errors in the energy to predict as best as possible the measured temperatures.

Period 4 (fixed set point at 25 °C): The MAE and ρ of the comparison of the energy consumed by the energy model and the real energy required by the households are shown for a set point of temperature established at 25 °C in Figure 10 and Figure 11, respectively. Two and one outliers, respectively, are not shown in the figures because they were not representative of the models.

The MAE in base model oscillated in performance from Q4 to Q1, but was improved in all cases by the calibration process (red and sky-blue), except in the living room of both houses where the unique model was ranked Q3, worse than the base model (Q1). For ρ, all the models performed similarly. In relation to the distance between red and sky-blue, we observed an anomaly in the living room of both houses because the unique model (sky-blue) performed even worse than the base model (dark blue). This is the only place where this happened; in the other thermal zones, the pattern was similar. In relation to the position in the contest, the red model, again, was ranked first in MAE, and for ρ, the values were similar for all the models.

As in Figure 6 and Figure 12 show the MAE produced by comparing the indoor temperatures of the energy models with the interior temperatures of the houses in the period of constant set point at 30 °C in the living space.

The models created for the experiment, as mentioned in previous paragraphs, do not produce any uncertainty error when representing the temperature of the building. The models provide the energy consumed when representing the interior temperature of the zones, as seen in the previous figure (Figure 12).

Period 5 (free oscillation): The MAE and ρ of the comparison of the interior temperatures reached by the energy models and the real indoor temperatures of the dwelling, a period of free oscillation, are shown where there is no artificial energy input to the buildings, except for the heating produced by the weather, in Figure 13 and Figure 14, respectively. Four outliers are not shown in Figure 14 because they were not representative of the models.

In this case, the MAE of the base model was always improved except in the kitchen and bedroom of houseO5. ρ was slightly improved but the margin was low because all the models produced good results for this index. The distance between the unique model (sky-blue) and the best model of the period (red) in MAE was small; they were always in the same quartile. The same occurred for ρ, except in the kitchen of house N2, where the sky-blue model was slightly better than red, which was unusual. With respect to the position of the red and sky-blue models in the contest, the leading position was unclear; red was the best in four out of eight in the Figure 13, and both models were always in Q1 except for the kitchen in house N2. Similar results were obtained in the Figure 14.

Once the results of the models were analyzed and compared with those of the contest, it was decided to check the models of both houses in different periods (checking periods)in order to evaluate their robustness (Table 4 and Table 5). As an example, the model calibrated in Period 2 of house N2 was also simulated in Periods 3, 4, and 5. It should be remembered that Period 1 is an initialization period and therefore it has not been taken into account in the calibration processes. At the same time, new models have been introduced for this demonstration, calibrated in several periods at the same time and tested in the rest, to see if the combination produced better results in the models, these models are:

For the N2 house:

- P 3-4 5: model adjusted in Periods 3, 4, and 5 and checked in Period 2.
- P 3-4: model adjusted in Periods 3 and 4 and checked in Period 2.
- P 2-4-5: model adjusted in Periods 2, 4, and 5 and checked in Period 3.
- P 2-3-5: model adjusted at Periods 2, 3, and 5 and checked at Period 4.
- P 2-3-4: model fitted at Periods 2, 3, and 4 and checked at Period 5.
- P 3-4: model fitted in Periods 3 and 4 and checked in Period 5.

For the O5 house:

- P 3-4 5: model adjusted in Periods 3, 4, and 5 and checked in Period 2.
- P 3-5: model adjusted in Periods 3 and 4 and checked in Period 2.
- P 2-4-5: model adjusted in Periods 2, 4, and 5 and checked in Period 3.
- P 2-3-5: model adjusted at Periods 2, 3, and 5 and checked at Period 4.
- P 3-5: model adjusted in Periods 3 and 4 and checked in Period 4.
- P 2-3-4: model fitted in Periods 2, 3, and 4 and checked in Period 5.

Table 4 and Table 5 show the results obtained. The models have been ordered according to their adjustment to the real data, showing first of each period, the model that has the best fit with reality and so on.

Analyzing the data the first observation that can be made is that models calibrated in their periods are the best for that period. Secondly as a general rule models calibrated in energy periods, are the best when they are checked in energy periods (2 and 4) and models calibrated in temperature periods are the best when they are checked in temperature periods (3 and 5). Thirdly the models that use the whole data available perform better than models that use mixed data (energy and temperature) and three o two periods. Finally, models that used mixed data perform better in checking period that models calibrated in temperature period and checked in energy period and vice-verse.

The uncertainty indices proposed by the designers of the Annex 58 experiment to assess the degree of fit of the models were MAE and ρ. In this study, the indexes from the available standards (ASHRAE Guidelines 14, IPMVP, and FEMP) were used because they offer a reference of quality. Table 6 provides a compilation of all the indexes produced by the energy models for all the zones analyzed (living room, children’s room, bedroom, and kitchen). All the calibrated models met the higher standard (IPMVP) for energy and temperature, and, despite the base model being categorized as good quality, the calibration process increased the quality.

The goal of the calibration process was to adjust the energy models to reality so that reliably depict the real building. Table 7 lists the parameters that best represent reality for each energy model studied. This information could be used to replicate the whole experiment, for confirmation of the results by others researchers, or to improve those results. In the original experiment, there was no information about the parameters of the model, which complicates the performance of comparative tests.

**Figure 15:**
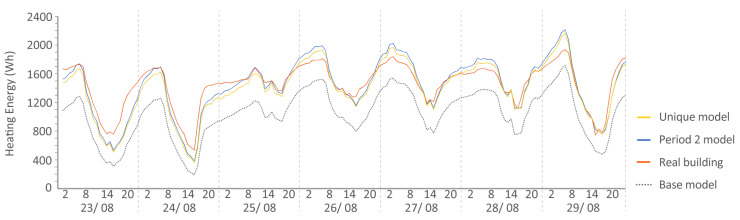
Energy consumed by the N2 house. Period 2 (set point at 30 ${}^{{}^{\circ}}$C). Period 2 model: Coefficient of Variation of Mean Square Error (CV(RMSE)): 9.06% Normalized Mean Bias Error (NMBE): 0.02% R^2^: 92.70%. Base model: CV(RMSE): 29.05% NMBE: 0.21% R^2^: 91.93%. Unique model: CV(RMSE): 9.65% NMBE: 0.04% R^2^: 91.80%.

**Figure 16:**
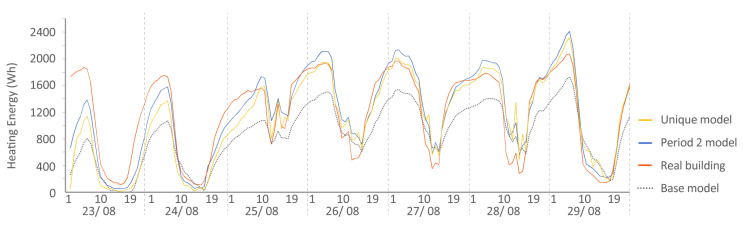
Energy consumed by house O5, Period 2 (set point at 30 ${}^{{}^{\circ}}$C). Period 2 model: CV(RMSE): 21.75%, NMBE: 0.02%, and R^2^: 83,78%. Base model: CV(RMSE): 39.77%, NMBE: 0.24%, and R^2^: 69.18%. Unique model: CV(RMSE): 28.41%, NMBE: 0.02%, and R^2^: 74.18%.

**Figure 17:**
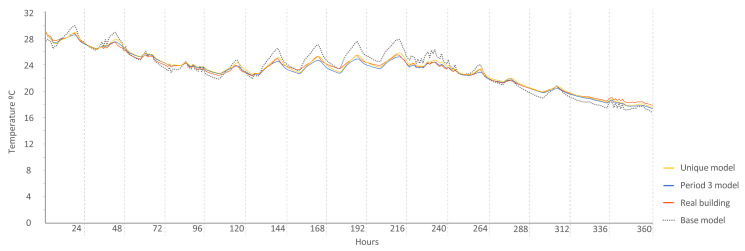
Temperature in house N2, Period 3 (ROLBS). Period 3 model: CV(RMSE): 1.24%, NMBE: 0.49%, and R^2^: 99.09%. Base model: CV(RMSE): 3.71%, NMBE: −0.93%, and R^2^: 95.45%. Unique model: CV(RMSE): 1.23%, NMBE: −0.38%, and R^2^: 99.07%.

**Figure 18:**
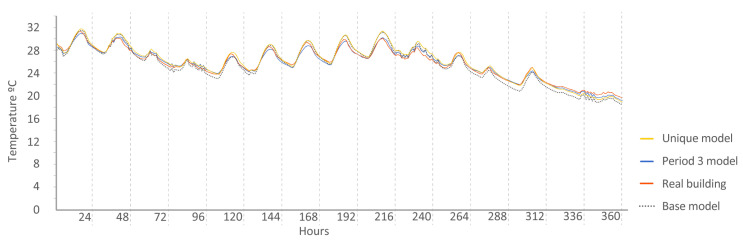
Temperature in house O5, Period 3 (ROLBS). Period 3 model: CV(RMSE): 1.26%, NMBE: 0.13%, and R^2^: 98.69%. Base model: CV(RMSE): 2.51%, NMBE: 0.83%, and R^2^: 98.73%. Unique model: CV(RMSE): 2.19%, NMBE: −0.97%, and R^2^: 98.59%.

**Figure 19:**
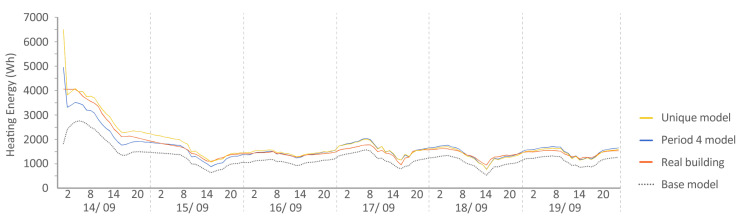
Energy consumed by house N2, Period 4 (set point at 25 ${}^{{}^{\circ}}$C). Period 4 model: CV(RMSE): 11.76%, NMBE: −0.05%, and R^2^: 91.70%. Base model: CV(RMSE): 30.98%, NMBE: 0.11%, and R^2^: 90.01%. Unique model: CV(RMSE): 14.46%, NMBE: −0.03%, and R^2^: 92.77%.

**Figure 20:**
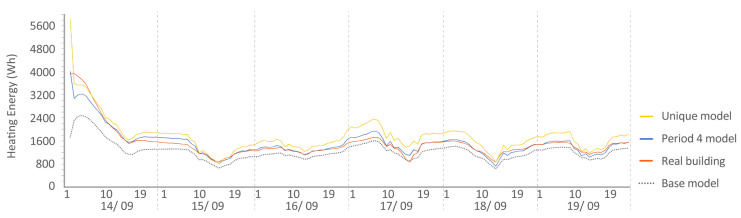
Energy consumed by house O5, Period 4 (set point at 25 ${}^{{}^{\circ}}$C). Period 4 model: CV(RMSE): 9.30%, NMBE: −0.01%, and R^2^: 94.36%. Base model: CV(RMSE): 25.78%, NMBE: −0.09%, and R^2^: 86.36%. Unique model: CV(RMSE): 21.12%, NMBE: −0.13%, and R^2^: 87.70%. It is remarkable the good performance of the base model in this case with a CV(RMSE) within ASHRAE standard. On some days, it improves the unique model.

**Figure 21:**
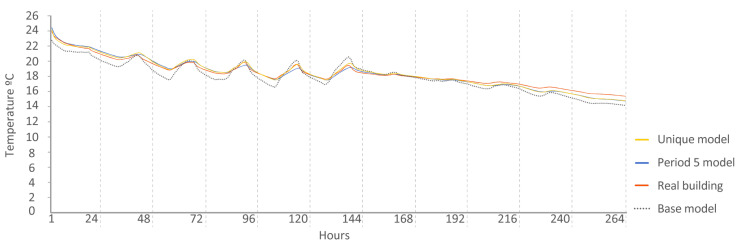
Temperature in house N2, Period 5 (free oscillation). Period 5 model: CV(RMSE): 1.67%, NMBE: 0.37%, and R^2^: 99.01%. Base model: CV(RMSE): 3.84%, NMBE: 2.52%, and R^2^: 92.43%. Unique model: CV(RMSE): 1.63%, NMBE: 0.11%, and R^2^: 98.66%.

**Figure 22:**
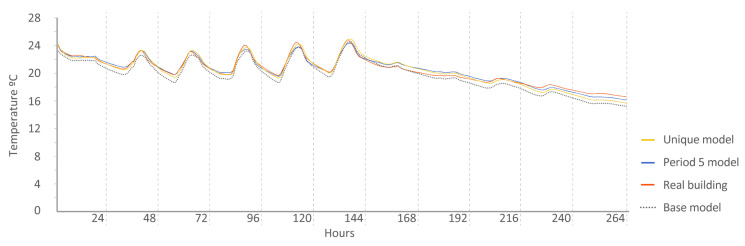
Temperature in house O5, Period 5 (free oscillation). Period 5 model: CV(RMSE): 1.57%, NMBE: −0.11%, and R^2^: 97.52%. Base model: CV(RMSE): 3.64%, NMBE: 3.06%, and R^2^: 97.75%. Unique model: CV(RMSE): 2.12%, NMBE: 0.36%, and R^2^: 97.75%.

The following Figure 15, Figure 16, Figure 17, Figure 18, Figure 19, Figure 20, Figure 21 and Figure 22 show the results of energy and temperature produced by the calibrated models (unique and period models) and the base model for each of the periods of the experiment in a temporal sequence. These data were compared with the real data measured in the original experiment. The graphs clearly visualize the degree of adjustment of the calibrated models and the unique model in each period, as well as how the calibration process captures the reality of the data produced by the energy model.

All the graphs shown for the different periods proposed by the experiment have elements in common. The most accurate models are those calibrated in each period. The unique model produced very satisfactory results, with its graph being similar to reality, but without improving the results of the calibrated model in its period. Finally, the model that performed the worst in all the periods was the base model, which emphasizes the importance of the calibration process.

By completing a global review of the results produced by the models provide in each validation period, we observed how both the model adjusted in its period and the unique model were among the best. Notably, the models adjusted for each period are those that produced the best results, always positioned better than the unique model. However, the unique model provides generality; it provides optimal results for all the proposed study periods.

In the paper that inspired this experiment [61], the large amount of data needed to construct optimal energy models that offer a good fit between reality and simulation is mentioned, “Similar data sets are needed from other, larger building types, but it would require a high level of resourcing to undertake such an experiment with a similar level of detail as the experiment described in this paper.” One of the achievements of the experiment was matching and, in many periods, improving the participants’ fitting data using only 52% of the sensors provided for the test. Not only is the sensor reduction remarkable but also the reduction of the most intrusive or expensive sensors. The experiment mainly used temperature measurement and energy measurement sensors, which may be the most economical and least intrusive. This considerably facilitated the process of adjusting the energy model, decreasing the cost of the data needed to perform this process while not affecting the operation of the building.

The adjustment process developed in this study is quick and simple, in which the energy model is found by adjusting four parameters present in the building, such as the experimental capacitance of the interior air, the internal mass, the infiltrations, and the thermal bridges. The right combination of these parameters produces an energy model that captures the thermal dynamics of the real building. The rest of the building materials do not suffer any variation in their characteristics. This methodology respects the building’s physics by generating energy models that adjust to the constructive reality of the buildings. Therefore, these are models that can be used by anyone with a minimum knowledge of construction since all the properties of the construction are those specified in the documents provided for the modeling process.

Many elements and people are involved in the process of building construction, which can produce discrepancies between the project or construction data and the real building data. For example, concrete is a material manufactured in a cement plant that is brought to the site. The installation process involves different factors that can vary its specifications, such as climate, installation methodology, etc. Gathering all the uncertainties that may arise in the construction phase is impractical. Some of them can be determined later, but the identification of most parameters is a task that cannot be assumed by modelers. In the adjustment process proposed in this paper, the construction specifications are entered into the model according to the documentation provided. The differences from reality can be assumed by the elements introduced in the calibration process (capacitances, internal mass, infiltrations, and thermal bridges) depending on the uncertainty of the model.

Some of these parameters, such as experimental capacitance and internal mass, are able to absorb the possible mismatches between measured and simulated data. The values used for feeding the algorithm are free; therefore, they do not have physical sense in some cases. We could consider these parameters as pure black box parameters. Thermal bridges and infiltration are a kind of mix. Thermal bridges oscillate around the values provided by the designers of the contest: 2.5 to 2.9 m^2^K/W. With a maximum value of five and a minimum value of zero, this constraint tries to give the parameter a physical sense; however, this parameter can be overfitted to match the measured values. For this reason, a comparison in a checking period should be conducted. This was one of the main problems with this experiment: a lack of verification periods with similar characteristics. For infiltration, the methodology used is the leakage area; this area is varied between 0 and 100 cm^2^ for each zone based on the authors’ previous modeling experience. This methodology gives the option for this parameter to be dynamically adjusted based on external conditions: wind speed, wind direction, and outdoor temperature. For this reason, infiltration has a lower possibility of overfitting. These elements can absorb the possible mismatches in the entered data, producing a result that could be considered a grey box model in which some parameters do not have a physical sense. However, these differences are not reflected in the documentation provided by the contest; without these adjustments, it would be unlikely that a high efficiency BEM could be constructed.

Two exercises were undertaken in this study: the adjustment of the model for each validation period and the construction of a high quality energy model for all the proposed periods. Notably, overfitting can occur during the calibration processes. This over-adjustment may be influenced by various factors, such as the weather and the indoor conditions in the thermal zone. Each energy model was adapted to the conditions provided in its period, such as the interior temperature or the energy consumed. For a model to be viable in a period other than its adjustment stage, the objective functions created for its convergence with the reality of the different spaces must have a correlation. If this correlation is weak or does not exist, the model will not be able to reflect reality in other periods. However, the longer the adjustment period, the better the results produced by the model over time because it will identify the differences that may occur in the thermodynamic conditions of the building. If the period selected for the adjustment is short but reflects a high percentage of the average thermal reality of the building, the resulting model will be able to support other periods.

## 4. Conclusions

In this study, we used the data provided in Annex 58 for the validation of an adjustment methodology where the calibration model is based on using fewer, less intrusive, and low-cost sensors. The results obtained by the 21 participants in the study were used to conduct a comparative test of the methodology. The results were reported using the uncertainty indexes proposed by the ASHRAE, IPMVP, and FEMP standards to enable empirical validation.

The dataset provided in Annex 58 includes almost two months of data. The meteorological data supplied are continuous for this period, and, although the sensors’ data for the houses had some small gaps, this was easily solved through a process of interpolation. The experiments and data provided were the same for both houses.

The experiments had to be completed in the summer months, which produces certain limitations when adjusting the models. Since a limited amount of data is available, some aspects, such as the behavior of the model in the face of abnormal climate changes, such as heat or cold waves, may produce discrepancies in the results, as no similar reference has been introduced to the model that could provide information on the behavior of the building in these special cases. The model learns from the data provided to it. The process of adjusting a building will be more successful as long as the data provided includes as many singularities as possible, regardless of the duration of the data.

The ability to adjust the envelope in periods with a wide range of temperature differences could not be tested. This also led to the energy input to the houses being limited, with only mechanical ventilation being tested. House occupation was excluded from the process to simplify the test. The period of data provided is relatively brief, and we would have preferred having a validation period independent of the calibration phases to more robustly verify the reliability of the models over time.

The two months of data were divided into five periods to test the strength of the calibration process in different circumstances. To complete the experiment, we performed two types of energy model calibration: the creation of a calibrated model for each proposed period and a calibration together with all the periods united (unique model).

The results demonstrated that the methodology used for the resolution of the proposed problem was valid. The models were adjusted to the requirements proposed in the Annex 58 exercise by reducing the sensors required by 47.5% and by not using any of the tests and essays provided for the resolution of the experiment.

The most accurate models were those that were calibrated in each period, but the results in the joint calibration showed that the methodology is robust, since they were also among the most accurate of the set of models produced by the experiment

Analyzing the results in all periods, the models calibrated with the proposed methodology produced the best results globally. Even the unique model, calibrated considering all five periods, produced results well above the average for the exercise.

With this analysis, we demonstrated that the proposed methodology for the calibration of energy models precise reproduces the thermal and energetic reality of the building while significantly reducing the number of sensors needed to produce optimal results. This led to a substantial reduction in costs, both in terms of human resources and equipment, as well as in the economics of constructing energy models, thus increasing the accessibility of these models for the building market.

## Figures and Tables

**Table 1:** Sensors provided by the Annex 58 experiment. Grey indicates the sensors used.

House	Location	Sensor	Units	Measured
N2/O5	doorway	Temperature	°C	Indoor air
N2/O5	bedroom	Temperature	°C	Indoor air
N2/O5	bath	Temperature	°C	Indoor air
N2/O5	child room	Temperature	°C	Indoor air
N2/O5	living h125 cm	Temperature	°C	Indoor air
N2/O5	kitchen	Temperature	°C	Indoor air
N2/O5	corridor	Temperature	°C	Indoor air
N2/O5	living h67 cm	Temperature	°C	Indoor air
N2/O5	living h187 cm	Temperature	°C	Indoor air
N2/O5	west facade S BL1	Temperature	°C	Surface temperature
N2/O5	west facade S ES	Temperature	°C	Surface temperature
N2/O5	west facade S IS	Temperature	°C	Surface temperature
N2/O5	attic east	Temperature	°C	Indoor temperature
N2/O5	attic west	Temperature	°C	Indoor air
N2/O5	vent SUA	Temperature	°C	Ventilation temperature
N2/O5	vent ODA	Temperature	°C	Ventilation temperature
N2/O5	vent EHA	Temperature	°C	Ventilation temperature
N2/O5	cellar	Temperature	°C	Indoor air
N2/O5	child’s room	Electrical power	W	Heating power
N2/O5	living room	Electrical power	W	Heating power
N2/O5	kitchen	Electrical power	W	Heating power
N2/O5	bathroom	Electrical power	W	Heating power
N2/O5	bedroom	Electrical power	W	Heating power
N2/O5	doorway	Electrical power	W	Ventilation power
N2/O5	vent EHA fan	Electrical power	W	Ventilation power
N2/O5	vent SUA fan	Electrical power	W	Ventilation power
N2/O5	vent EHA VFR	Air speed	m^3^/h	Ventilation air speed
N2/O5	vent SUA VFR	Air speed	m^3^/h	Ventilation air speed
N2/O5	vent thP	Electrical power	W	Thermal power
N2	west facade S BL1	Heat flux	W/m^2^	Heat flux
N2	west facade S IS	Heat flux	W/m^2^	Heat flux
O5	west facade S BL1	Heat flux	W/m^2^	Heat flux
O5	west facade S IS	Heat flux	W/m^2^	Heat flux
N2	N2 living rH h 125 cm	Humidity	%	Relative humidity
O5	N2 living rH h 125 cm	Humidity	%	Relative humidity
N2/O5	Weather station	Wind speed	m/s	Wind speed
N2/O5	Weather station	Wind direction	º	Wind direction
N2/O5	Weather station	Relative humidity	%	Relative humidity
N2/O5	Weather station	Radiation	W/m^2^	Vertical radiation south
N2/O5	Weather station	Radiation	W/m^2^	Global radiation
N2/O5	Weather station	Radiation	W/m^2^	Diffuse radiation
N2/O5	Weather station	Radiation	W/m^2^	Vertical radiation north
N2/O5	Weather station	Radiation	W/m^2^	Vertical radiation east
N2/O5	Weather station	Radiation	W/m^2^	Vertical radiation west
N2/O5	Weather station	Temperature	°C	Ambient temperature
N2/O5	Weather station	Temperature	°C	Ground temperature 0 cm.
N2/O5	Weather station	Temperature	°C	Ground temperature 50 cm.
N2/O5	Weather station	Temperature	°C	Ground temperature 100 cm.
N2/O5	Weather station	Temperature	°C	Ground temperature 200 cm.
N2/O5	Weather station	Radiation	W/m^2^	Long-wave radiation horizontal
N2/O5	Weather station	Radiation	W/m^2^	Long-wave radiation west

**Table 2:** Data periods, data provided, and data requested.

Period	Date	Configuration	Data Provided	Data Requested
Period 1	2013/8/21 to 2013/8/23	Initialization (constant temperature)	Temperature and heat inputs	-
Period 2	2013/8/23 to 2013/8/30	Constant temperature (nominal 30 °C)	Temperature and heat inputs	Heat outputs
Period 3	2013/8/30 to 2013/9/14	ROLBS heat inputs in living room	Temperature and heat inputs	Temperature outputs
Period 4	2013/9/14 to 2013/9/20	Re-initialization Constant temp.	Temperature and heat inputs	Heat outputs
Period 5	2013/9/20 to 2013/9/30	(nominal 25 °C) Free float	Temperature inputs	Temp. outputs

**Table 3:** Calibration criteria of Federal Energy Management Program (FEMP), American Society of Heating, Refrigerating and Air-Conditioning Engineers (ASHRAE), and International Performance Measurement and Verification Protocol (IPMVP).

Data Type	Index	FEMP 3.0 Criteria	ASHRAE G14-2002	IPMVP
**Calibration Criteria**				
Monthly Criteria %	NMBE	±5	±5	±20
	CV(RMSE)	±15	±15	-
Hourly Criteria %	NMBE	±10	±10	±5
	CV(RMSE)	±30	±30	±20

**Table 4:** Results of the N2 house models in the checking periods.

			MAE Index				ρ Index			
House	Calibrated Period	Checking Period	Living Room	Children’s Room	Bedroom	Kitchen	Living Room	Children’s Room	Bedroom	Kitchen
			W/h	W/h	W/h	W/h	%	%	%	%
N2	Period 2	Period 2	51	29	21	25	97.3	63.9	91.7	87.9
N2	Period 4	Period 2	52	35	22	30	97.2	55.8	91.9	87.6
N2	Unique model	Period 2	69	81	24	25	96.2	58.9	91.1	87.7
N2	P 3-4	Period 2	52	112	48	26	97.2	59.4	91.5	87.5
N2	Period 5	Period 2	85	132	22	81	96.2	56.1	91.2	74.1
N2	P 3-4-5	Period 2	81	101	37	26	96.3	59.4	91.6	87.5
N2	Period 3	Period 2	211	194	45	102	96.3	58.1	91.3	71.6
N2	Period 4	Period 4	56	37	37	34	97.8	59.7	85.7	82.2
N2	Period 2	Period 4	67	169	32	41	94.8	81.5	86.9	85.1
N2	Unique model	Period 4	209	77	43	40	94.6	67.8	85.4	86.1
N2	P 2-3-5	Period 4	209	91	43	47	92.3	94.0	55.1	85.1
N2	Period 3	Period 4	318	153	46	80	94.4	62.4	86.9	67.2
N2	Period 5	Period 4	242	352	45	170	94.8	32.8	87.7	37.2
			°C	°C	°C	°C	%	%	%	%
N2	Period 3	Period 3	0.32	0.23	0.22	0.55	98.5	97.5	97.6	91.7
N2	Unique model	Period 3	0.40	0.33	0.26	0.58	98.9	95.6	97.3	96.7
N2	Period 5	Period 3	0.36	0.27	0.34	0.59	98.5	97.2	97.3	92.0
N2	P 2-4-5	Period 3	0.54	0.97	0.29	0.58	98.9	86.6	97.1	96.6
N2	Period 4	Period 3	1.02	1.04	1.04	0.86	98.4	97.3	95.4	95.5
N2	Period 2	Period 3	1.20	1.05	0.91	0.79	97.6	85.8	95.4	96.4
N2	Period 5	Period 5	0.34	0.22	0.21	0.51	99.5	99.9	99.0	94.6
N2	Unique model	Period 5	0.30	0.26	0.21	0.59	99.7	99.9	99.1	95.4
N2	Period 3	Period 5	0.40	0.28	0.23	0.51	99.6	99.8	99.1	94.8
N2	P 3-4	Period 5	1.11	0.25	0.26	0.65	97.0	99.8	98.6	95.7
N2	P 2-3-4	Period 5	1.14	0.22	0.32	0.73	96.1	99.6	98.1	88.9
N2	Period 4	Period 5	1.31	0.52	0.88	0.86	96.7	99.5	97.0	95.1
N2	Period 2	Period 5	1.42	1.55	0.78	0.81	94.6	98.8	96.8	95.3

**Table 5:** Results of the O5 house models in the checking periods.

			MAE Index				ρ Index			
House	Calibrated Period	Checking Period	Living Room	Children’s Room	Bedroom	Kitchen	Living Room	Children’s Room	Bedroom	Kitchen
			W/h	W/h	W/h	W/h	%	%	%	%
O5	Period 2	Period 2	117	40	26	29	92.4	84.1	73.8	92.1
O5	Period 4	Period 2	117	42	27	30	92.0	83.5	72.3	91.6
O5	Unique model	Period 2	146	53	33	34	87.1	82.8	86.1	88.3
O5	P 3-4-5	Period 2	154	82	35	43	85.8	79.2	83.9	85.2
O5	P 3-5	Period 2	199	109	38	38	87.6	78.1	82.5	89.1
O5	Period 5	Period 2	219	87	29	59	88.8	76.5	84.1	87.4
O5	Period 3	Period 2	197	122	63	66	84.4	78.8	75.3	82.6
O5	Period 4	Period 4	51	31	13	19	97.4	51.9	97.0	96.2
O5	Unique model	Period 4	182	48	31	26	97.3	37.5	96.3	96.8
O5	Period 3	Period 4	128	70	52	42	95.8	29.1	96.9	95.5
O5	P 2-3-5	Period 4	294	55	50	65	96.4	33.7	95.0	95.7
O5	P 3-5	Period 4	399	99	38	65	96.2	41.5	90.5	95.7
O5	Period 2	Period 4	143	70	112	31	94.1	16.8	68.5	89.9
O5	Period 5	Period 4	457	76	43	126	96.1	36.9	82.3	91.3
			°C	°C	°C	°C	%	%	%	%
O5	Period 3	Period 3	0.45	0.29	0.19	0.28	98.5	99.0	98.8	98.7
O5	Unique model	Period 3	0.79	0.36	0.22	0.35	98.6	98.4	98.4	98.8
O5	P 2-4-5	Period 3	0.90	0.44	0.22	0.35	98.6	98.2	98.4	98.7
O5	Period 5	Period 3	0.46	0.53	0.30	0.47	97.9	98.8	99.3	95.8
O5	Period 4	Period 3	0.64	0.92	0.54	0.48	97.7	92.9	95.5	98.8
O5	Period 2	Period 3	1.78	1.12	1.22	1.25	98.3	98.2	88.4	96.2
O5	Period 5	Period 5	0.39	0.13	0.18	0.27	98.3	99.5	99.4	98.6
O5	Period 3	Period 5	0.66	0.34	0.20	0.30	98.3	99.6	99.1	99.3
O5	Unique model	Period 5	0.52	0.27	0.19	0.35	98.1	99.2	99.3	98.6
O5	P 2-3-4	Period 5	0.81	0.25	0.24	0.42	97.9	98.9	98.9	98.2
O5	Period 4	Period 5	0.60	1.23	0.58	0.41	97.9	96.4	99.4	97.6
O5	Period 2	Period 5	1.60	0.63	1.35	1.24	97.3	97.8	99.5	93.4

**Table 6:** Compilation of results for dwellings N2 and O5. ■ Thermal zones that satisfy the IPMVP standards. ■ Thermal zones that satisfy the ASHRAE and FEMP standards. ■ Thermal zones that do not satisfy the IPMVP, ASHRAE, and FEMP standards.

			MAE		CV(RMSE)		NMBE		R^2^		ρ	
Houses	Periods	Models	Temp. °C	Energy W/h	Temp. %	Energy %	Temp. %	Energy %	Temp. %	Energy %	Temp. %	Energy %
N2	Period 2 (Set point 30 °C)	Period 2 model	0	104	0%	9.06%	0%	0.02%	100%	92.70%	100%	96.89%
N2	Period 2 (Set point 30 °C)	Unique model	0	111	0%	9.65%	0%	0.04%	100%	91.80%	100%	96.12%
N2	Period 2 (Set point 30 °C)	Base model	0	410	0%	29.05%	0%	0.21%	100%	91.93%	100%	96.58%
O5	Period 2 (Set point 30 °C)	Period 2 model	0	197	0%	21.75%	0%	0.02%	100%	83.78%	100%	92.24%
O5	Period 2 (Set point 30 °C)	Unique model	0	230	0%	28.41%	0%	0.02%	100%	74.18%	100%	87.64%
O5	Period 2 (Set point 30 °C)	Base model	0	396	0%	39.77%	0%	0.24%	100%	69.18%	100%	86.92%
N2	Period 3 (ROLBS)	Period 3 model	0.23	0	1.24%	0%	0.49%	0%	99.09%	0%	98.79%	100%
N2	Period 3 (ROLBS)	Unique model	0.24	0	1.23%	0%	−0.38%	0%	99.07%	0%	98.97%	100%
N2	Period 3 (ROLBS)	Base model	0.69	0	3.71%	0%	−0.93%	0%	95.45%	0%	97.84%	100%
O5	Period 3 (ROLBS)	Period 3 model	0.28	0	1.26%	0%	0.13%	0%	98.69%	0%	99.09%	100%
O5	Period 3 (ROLBS)	Unique model	0.45	0	2.19%	0%	−0.97%	0%	98.59%	0%	99.02%	100%
O5	Period 3 (ROLBS)	Base model	0.55	0	2.51%	0%	0.83%	0%	98.73%	0%	99.13%	100%
N2	Period 4 (Set point at 25 °C)	Period 4 model	0	131	0%	11.76%	0%	−0.05%	100%	91.70%	100%	96.30%
N2	Period 4 (Set point at 25 °C)	Unique model	0	122	0%	14.46%	0%	−0.03%	100%	92.77%	100%	96.90%
N2	Period 4 (Set point at 25 °C)	Base model	0	432	0%	30.98%	0%	0.11%	100%	90.01%	100%	96.70%
O5	Period 4 (Set point at 25 °C)	Period 4 model	0	86	0%	9.30%	0%	−0.01%	100%	94.36%	100%	96.35%
O5	Period 4 (Set point at 25 °C)	Unique model	0	256	0%	21.12%	0%	−0.13%	100%	87.70%	100%	96.93%
O5	Period 4 (Set point at 25 °C)	Base model	0	276	0%	25.78%	0%	0.09%	100%	86.36%	100%	97.24%
N2	Period 5 (Free oscillation)	Period 5 model	0.25	—	1.67%	—	0.37%	—	99.01%	—	99.60%	—
N2	Period 5 (Free oscillation)	Unique model	0.24	—	1.63%	—	0.11%	—	98.66%	—	99.80%	—
N2	Period 5 (Free oscillation)	Base model	0.61	—	3.84%	—	2.52%	—	92.43%	—	96.50%	—
O5	Period 5 (Free oscillation)	Period 5 model	0.27	—	1.57%	—	-0.11%	—	97.52%	—	99.00%	—
O5	Period 5 (Free oscillation)	Unique model	0.35	—	2.12%	—	0.36%	—	97.25%	—	98.60%	—
O5	Period 5 (Free oscillation)	Base model	0.65	—	3.64%	—	3.06%	—	97.75%	—	98.80%	—

**Table 7:** Parameters selected in the calibration process for each energy model.

Houses	Object	Room	Period 2 Model	Period 3 Model	Period 4 Model	Period 5 Model	Unique Model	Base Model	Base Model	Unique Model	Period 5 Model	Period 4 Model	Period 3 Model	Period 2 Model	Room	Object	Houses
N2	Capacitance (Temp. capacity multiplayer)	Living room	10	10	10	20	10	-	-	10	10	10	2	0	Living room	Capacitance (Temp. capacity multiplayer)	O5
		Children’s room	20	1	40	10	20	-	-	20	1	20	5	1	Children’s room		
		Kitchen	20	140	1	130	10	-	-	20	20	10	5	1	Kitchen		
		Bedroom	10	40	1	50	50	-	-	40	60	10	15	5	Bedroom		
N2	Infiltrations (cm^2^)	Living room	0	0	0	0	1	-	-	70	100	0	0	10	Living room	Infiltrations (cm^2^)	O5
		Children’s room	25	25	25	0	25	-	-	40	50	40	10	75	Children’s room		
		Kitchen	0	0	0	0	0	-	-	0	20	0	0	1	Kitchen		
		Bedroom	0	0	1	0	0	-	-	0	20	0	10	20	Bedroom		
N2	Thermal bridge Partition/Floor (m^2^K/W)	Living room	0.001	0.001	0.001	0.001	0.001	2.940	2.940	2.940	2.940	2.940	2.940	2.940	Living room	Thermal bridge Partition/Floor (m^2^K/W)	O5
		Children’s room	0.001	0.001	0.001	0.001	0.001	2.940	2.940	0.001	0.001	0.001	0.001	0.001	Children’s room		
		Kitchen	0.001	0.001	0.001	0.001	0.001	2.940	2.940	2.940	2.940	2.940	2.940	2.940	Kitchen		
		Bedroom	0.001	0.001	0.001	0.001	0.001	2.940	2.940	0.200	0.200	0.200	0.200	0.200	Bedroom		
N2	Thermal bridge Partition/Ceiling (m^2^K/W)	Living room	0.001	0.001	0.001	0.001	0.001	2.940	2.940	2.940	2.940	2.940	2.940	2.940	Living room	Thermal bridge Partition/Ceiling (m^2^K/W)	O5
		Children’s room	0.001	0.001	0.001	0.001	0.001	2.940	2.940	0.001	0.001	0.001	0.001	0.001	Children’s room		
		Kitchen	0.001	0.001	0.001	0.001	0.001	2.940	2.940	2.940	2.940	2.940	2.940	2.940	Kitchen		
		Bedroom	0.001	0.001	0.001	0.001	0.001	2.940	2.696	0.001	0.001	0.001	0.001	0.001	Bedroom		
N2	Thermal bridge Wall/Ceiling (m^2^K/W)	Living room	3.000	0.001	3.000	1.000	3.000	2.696	2.696	1.000	0.001	5.000	2.000	1.000	Living room	Thermal bridge Wall/Ceiling (m^2^K/W)	O5
		Children’s room	0.001	2.500	0.001	2.000	0.001	2.696	2.696	0.001	5.000	0.001	0.001	1.000	Children’s room		
		Kitchen	0.001	0.001	0.001	0.001	0.500	2.696	2.696	1	0.001	0.001	1.000	0.001	Kitchen		
		Bedroom	0.001	0.500	0.100	1.000	0.001	2.696	2.450	0.001	1.000	0.001	1.000	0.001	Bedroom		
N2	Thermal bridge Wall/Floor (m^2^K/W)	Living room	2.000	0.001	2.000	0.100	2.000	2.450	2.450	1.000	0.001	5.000	0.001	0.001	Living room	Thermal bridge Wall/Floor (m^2^K/W)	O5
		Children’s room	0.001	1.500	0.100	2.000	0.100	2.450	2.450	1.000	2.000	5.000	5.000	0.001	Children’s room		
		Kitchen	0.100	0.001	0.100	0.001	0.100	2.450	2.450	0.001	0.001	0.001	0.001	1.000	Kitchen		
		Bedroom	0.100	1.500	0.100	0.100	0.100	2.450	2.261	0.001	0.001	1.000	1.000	5.000	Bedroom		
N2	Thermal bridge Wall/Wall (m^2^K/W)	Living room	1.500	0.500	1.500	1.500	0.100	2.261	2.261	1.000	0.001	5.000	5.000	0.001	Living room	Thermal bridge Wall/Wall (m^2^K/W)	O5
		Children’s room	0.001	1.000	0.001	0.001	0.500	2.261	2.261	0.001	5.000	5.000	5.000	1.000	Children’s room		
		Kitchen	0.500	0.001	1.000	0.001	0.500	2.261	2.261	0.001	0.001	1.000	5.000	0.001	Kitchen		
		Bedroom	0.100	0.001	0.100	0.001	0.500	2.261	2.261	1.000	1.000	1.000	1.000	5.000	Bedroom		
N2	Internal mass (m^2^)	Living room	10	80	1	70	70	-	-	30	80	20	43	0	Living room	Internal mass (m^2^)	O5
		Children’s room	10	1	90	1	10	-	-	20	0	50	0	0	Children’s room		
		Kitchen	0	0	0	0	0	-	-	10	30	5	5	0	Kitchen		
		Bedroom	0	0	0	0	0	-	-	10	5	20	1	70	Bedroom		

## Abbreviations

The following abbreviations are used in this manuscript:

IEA	International Energy Agency
SABINA	SmArt BI-directional multi eNergy gAteway
NFRC	National Fenestration Rating Council
EU	European Union
DOE	Department of Energy
BEM	Building Energy Model
NSGA-II	Non-Dominated Sorting Genetic Algorithm
IBP	Institute for Building Physics
HVAC	Heating Ventilation Air Conditioning
ROLBS	Randomly Ordered Logarithmic Binary Sequence
MAE	Mean Absolute Error
ρ	Spearman’s Rank Correlation Coefficient
ASHRAE	American Society of Heating, Refrigerating and Air-Conditioning Engineers
IPMVP	International Performance Measurement and Verification Protocol
FEMP	Federal Energy Management Program
CV(RMSE)	Coefficient of Variation of Mean Square Error
RMSE	Root Mean Square Error
*r*	Pearson Correlation Coefficient
R^2^	Square Pearson Correlation Coefficient
NMBE	Normalize Mean Bias Error
BE	Bias Error
*h*	Height
BL1	Boundary layer between insulation and brick wall rendering
ES	External Surface
IS	Internal Surface
SUA	Supply Air
ODA	Fresh Air
EHA	Exhaust Air
VFR	Volume flow rate
tpH	Thermal power
rH	Relative humidity
S	South
Temp.	Temperature
°C	Celsius degrees
W	Watt
%	Percentage
m^3^/h	Cubic meters per hour
w/m^2^	watt to square meter
m/s	meter per second
cm	centimeter
m	meter
Q	quartile
BMS	Building Management System
